# Using a Hazard Quotient to Evaluate Pesticide Residues Detected in Pollen Trapped from Honey Bees (*Apis mellifera*) in Connecticut 

**DOI:** 10.1371/journal.pone.0077550

**Published:** 2013-10-15

**Authors:** Kimberly A. Stoner, Brian D. Eitzer

**Affiliations:** 1 Department of Entomology, The Connecticut Agricultural Experiment Station, New Haven, Connecticut, United States of America; 2 Department of Analytical Chemistry, The Connecticut Agricultural Experiment Station, New Haven, Connecticut, United States of America; Ecole des Mines d'Alès, France

## Abstract

Analysis of pollen trapped from honey bees as they return to their hives provides a method of monitoring fluctuations in one route of pesticide exposure over location and time. We collected pollen from apiaries in five locations in Connecticut, including urban, rural, and mixed agricultural sites, for periods from two to five years. Pollen was analyzed for pesticide residues using a standard extraction method widely used for pesticides (QuEChERS) and liquid chromatography/mass spectrometric analysis. Sixty pesticides or metabolites were detected. Because the dose lethal to 50% of adult worker honey bees (LD_50_) is the only toxicity parameter available for a wide range of pesticides, and among our pesticides there were contact LD_50_ values ranging from 0.006 to >1000 μg per bee (range 166,000X), and even among insecticides LD_50_ values ranged from 0.006 to 59.8 μg/bee (10,000X); therefore we propose that in studies of honey bee exposure to pesticides that concentrations be reported as Hazard Quotients as well as in standard concentrations such as parts per billion. We used both contact and oral LD_50_ values to calculate Pollen Hazard Quotients (PHQ = concentration in ppb ÷ LD_50_ as μg/bee) when both were available. In this study, pesticide Pollen Hazard Quotients ranged from over 75,000 to 0.01. The pesticides with the greatest Pollen Hazard Quotients at the maximum concentrations found in our study were (in descending order): phosmet, Imidacloprid, indoxacarb, chlorpyrifos, fipronil, thiamethoxam, azinphos-methyl, and fenthion, all with at least one Pollen Hazard Quotient (using contact or oral LD_50_) over 500. At the maximum rate of pollen consumption by nurse bees, a Pollen Hazard Quotient of 500 would be approximately equivalent to consuming 0.5% of the LD_50_ per day. We also present an example of a Nectar Hazard Quotient and the percentage of LD_50_ per day at the maximum nectar consumption rate.

## Introduction

With the serious annual losses of managed honey bees every year since 2006 in the US [1] and in other countries around the world [2,3], the levels and routes of exposure of honey bees to pesticides have come under scrutiny. In a review of studies of pesticide residues from around the world [4], maximum levels of 130 pesticide residues were reported from samples of wax, honey, bees, and pollen, usually taken from inside the hive. While this is important information, it is difficult to evaluate the relative effects of different pesticides when their concentrations are presented without any measure of toxicity to honey bees. 

Fortunately, because honey bees have long been used as a representative of non-target beneficial insects by environmental agencies around the world, there are values for acute contact toxicity to worker honey bee adults, measured as the lethal dose for 50% of the test population (LD_50_), supplied by the registrants for nearly all pesticides used in the field. In the US, this information is publicly available in the Ecotoxicity Database of the Ecological Fate and Effects Division of Office Pesticide Programs of the US Environmental Protection Agency [5]. Another publicly available source, drawing on data from the European Union, is The Agritox Database of the Agence Nationale de Sécurité Sanitaire de l’Alimentation, de l’Environnement et du Travail in France [6]. In most cases, the LD_50_ values in these two databases are identical, but sometimes one database will have data not included in the other source. Neither database had LD_50_ values for coumaphos, or for the metabolites of imidacloprid, so these were obtained from published studies [7,8].

In the European Union, the risk posed by pesticides to honey bees is evaluated according to the European and Mediterranean Plant Protection Organization guidelines. These guidelines specify that moving from laboratory studies to semi-field studies depends on a trigger criterion, the Hazard Quotient (HQ = field application rate ÷ oral or contact LD_50_). When this criterion is greater than 50, semi-field studies are required [9,10]. We propose calculating a similar Pollen Hazard Quotient (PHQ), using the concentration of pesticide residue in pollen in the numerator instead of the field application rate, in order to be able to better evaluate the hazard from pesticide residues in pollen in relation to acute toxicity to honey bees. Using this same standard for all pollen data will enable more efficient initial screening for hazards.

When we provide beekeepers in our region with information about what pesticides the bees are bringing into the hive at different sites and over a period of years, the beekeepers need to be able to put those pesticide concentrations into a context of hazard to their bees, and PHQ values provide a step toward relating pesticide concentrations to acute toxicity to worker bees. Then the next step is to relate PHQ values back to a percentage of the LD_50_ consumed by the bees as pesticide residue in the pollen. Assuming a maximum level of pollen consumption of 9.5 mg of pollen per bee per day for adult nurse bees [11], a bee consuming pollen with a PHQ of 50 would be consuming approximately 0.05% of the LD_50_ rate per day during her period of maximum consumption. With the same assumption, a PHQ of 500 would correspond with 0.5% of the LD_50_ per day. By using these PHQ levels as screening criteria, we can present to the beekeepers how often the measured pesticide residues exceed those levels at each site.

## Materials and Methods

### Sample Collection

Pollen was collected using Sundance™ I bottom-mounted pollen traps (Ross Rounds, Albany, NY). These traps operate by forcing the foraging bees returning to the hive to enter through a coarse double-screen grid that removes most of the pollen pellets held in the pollen baskets on the rear legs of the bees [12]. The pollen drops into a drawer protected above by a wooden tray to keep out most debris from the hive, a finer mesh screen to keep the bees from being able to reach the pollen, and another fine mesh screen below to allow ventilation. The drawer opens to the back of the hive, allowing removal of the pollen without disturbing the colony. All pollen was collected from the trap twice weekly, with two samples put immediately into 50 ml centrifuge tubes, frozen upon return from the field, and held at -20° C until analysis. Pollen was collected from a single hive in the apiary unless the amount of pollen per sample decreased below sufficient levels for analysis (often due to swarming of the colony) or the health of the hive declined, and then the trap was moved to a new hive in the same apiary. 

### Apiary Sites and Management

The sites chosen for sampling did not have a history of problems with honey bee health. They were chosen to be broadly representative of a range of sites in our state. Apiaries were maintained either by the state apiarist of the Connecticut Agricultural Experiment Station or a cooperating beekeeper. Pollen was collected all five years in the two sites managed by the state apiarist, New Haven and Hamden. The New Haven apiary was on the roof of one of the Experiment Station buildings in an area of single-family houses with well-maintained landscaping, adjacent to a college and near several parks within the city. The Hamden apiary was at the Lockwood Farm, also belonging to the Connecticut Agricultural Experiment Station, which grows a wide diversity of vegetable, fruit, and tree crops. The surrounding area includes a sizable tree nursery adjacent to the farm, in addition to predominantly suburban single family houses. Pollen was collected from the hives of the cooperating beekeeper in 2007-2010 in Farmington, in a mixed-use area with a small pumpkin field immediately adjacent, with suburban houses, a plant nursery, and extensive privately managed agricultural fields nearby. Pollen was collected in Ellington in 2009 and 2010, at the request of the cooperating beekeeper, in a more rural area at a topsoil and compost processing center with extensive areas of early successional growth, forest and agricultural fields. The site in Cheshire was an orchard where the cooperating beekeeper brought in bees to pollinate apples and blueberries, and pollen was collected only during the pollination season in 2007 and 2009.

To manage mites, all hives in the Experiment Station apiaries were treated annually in early September with Apiguard (active ingredient: thymol; VITA [Europe] Limited, c/o Landis International, Inc. Valdosta, GA) according to label instructions. The cooperating beekeeper used formic acid for mite control beginning in 2005. None of the apiaries studied had been treated with coumaphos or fluvalinate for at least two years before the beginning of the study. Terramycin was used for control of American foulbrood and fumagillin for *Nosema* as needed.

### Chemical Analysis

To reduce the number of samples analyzed, pollen samples were composited in 2008-2010. Composite samples were generated from individual sites by combining equal amounts (when possible) of pollen from samples taken over a 10 day period (3 composites per month per site). After thorough mixing the composites were analyzed in the same manner as samples that had not been combined. 

#### Extraction

All samples were extracted using a modified version of the QuEChERS (for Quick, Easy, Cheap, Effective, Rugged and Safe) protocol [13]. This protocol has many versions depending on choices of dispersants (which ones to use), bulk addition vs. columns, and buffered versus unbuffered acetonitrile. Although the versions might differ slightly in the extraction efficiency for an individual pesticide there is no “best” single procedure for a wide range of pesticides. During the first year of the project we tried a couple of modifcations (sample size, buffered or not, etc.) and decided to settle with the following procedure. Pollen samples (approximately 5 g) were combined with water to a final volume of 15 mL. To this sample was added 100 ng of isotopically labeled (d-4) imidacloprid (Cambridge Isotope Laboratories) as an internal standard. The samples were combined with 15 mL of acetonitrile, 6 g magnesium sulfate and 1.5 g sodium acetate, and 150 ul of acetic acid. After shaking and centrifuging, 10 mL of the supernatant was combined with 1.5 g magnesium sulfate, 0.5 g PSA , 0.5 g C-18 silica and 2 mL toluene. The samples were shaken and centrifuged and 6 mL of the supernatant was concentrated to 1 mL for instrumental analysis.

#### Analysis

Extracts were analyzed with liquid chromatography/mass spectrometry/mass spectrometry (LC/MS/MS). From 2007 through 2009, the LC system was an Agilent 1100 LC; 6 μL of the extract was injected onto a Zorbax SB-C18, 2.1 x 150 mm, 5 micron column. The column is gradient eluted at 0.25 mL per minute from 12.5% methanol in water to 100% methanol. Both solvents have 0.1% formic acid added. In 2010, the LC system was replaced with an Agilent 1200 Rapid Resolution system using a Zorbax SB-C18 Rapid Resolution HT 2.1 x 50 mm, 1.8 micron column using a 3 μL injection with the gradient going from 5% methanol in water to 100 % methanol at 0.45 mL/min. In both years, the LC was coupled to a Thermo-LTQ, a linear ion trap mass spectrometer. The system is operated in the positive ion electrospray mode, with a unique scan function for each compound allowing for MS/MS monitoring. 

#### Quality Assurance

Samples were analyzed in batches of up to 20 samples. With each batch of samples a reagent blank sample and 1-3 duplicate spiked samples were analyzed. The spiked samples were prepared with various mixed pesticide samples and spiked into the pollen at concentrations in a range from 5 to 30 parts per billion. It should be noted that due to the wide number of pesticides analyzed not all pesticides were spiked along with each batch of samples. Detection limits were estimated by examining the peak to noise ratios in low level spiked peaks. The reported limits are based on the examination of a number of these spiked samples over the years, though there were some small variations from sample to sample. It should be noted that when the instrumentation was changed in 2010, which allowed us to analyze samples at a faster rate, there was a small loss in sensitivity for some pesticides. For consistency in the comparison between years, data are reported only when higher than the more conservative detection limit. Samples were quantified by use of the spiked internal standard but matrices on individual pesticides were not accounted for.

### Screening the Results for PHQ Levels

The data were screened using pre-determined PHQ levels to determine at how many sites and in how many detections at each site, the residues of each pesticide exceeded those levels. The PHQ level of 50 was chosen based on the history of use by the European Union. We also chose a PHQ of 500 as a level that we could relate to a percentage of the LD50 at a maximum daily rate of pollen consumption. We assumed that bees come in contact with or consume a certain quantity of pollen, calculated an estimated exposure, and then compared that estimate to the contact or oral LD_50_. Various estimates of daily pollen consumption have been made by previous investigators, depending on whether the starting point is the pollen consumption of the entire colony divided by an estimated number of worker bees [14] or starting from measurements of the maximum daily rate of pollen consumption of individual worker bees, which would be nurse bees in the first few days after emergence [15,16]. We used a value of 9.5 mg/bee/day, the maximum daily rate of pollen consumption of an individual nurse bee [11], in calculating daily exposure in relation to contact and oral LD_50_. A simple calculation showed that at this maximum rate of pollen consumption of pollen with a PHQ of 500, the bee would consume approximately 0.5% of the LD_50_ for the pesticide per day. This PHQ level was chosen for screening because it provides an easily understood relationship to the LD_50_ rate.

## Results and Discussion

Sixty pesticides, including some major metabolites of pesticides as well as active ingredients, were detected (Table 1). It should be noted that even using the specified sample cleanup, the pollen matrix remained highly complex; thus we chose only to use our available LC/MS-MS instrumentation for enhanced specificity in pesticide detection at very low concentrations, providing greater confidence in our data on pesticides detected during a multi residue screen. However, this choice precluded detection of some classes of pesticides (such as pyrethroid insecticides) which can require additional sample cleanup steps and gas chromotography/mass spectrometry (GC/MS) in electron impact or negative chemical ionization modes. Therefore we do not report the same set of pesticides as some previous studies on pesticide residues in pollen [17]. This procedure does not detect chlorothalonil, one of the more commonly reported pesticides in other studies. It should also be noted that the compositing of samples during 2008-2010 tends to smooth the data so that the highest concentrations may be somewhat reduced in those years relative to 2007 and 2011 when samples were not composited before analysis. In those years when we did not composite samples we found that there can be a great difference in concentration in samples taken just days apart. 

**Table 1:** Pesticides detected in pollen trapped by honey bee hives, with pesticide use and information on acute toxicity to adult worker honey bees.

**Pesticide**	**Use**	**Contact LD_50_ ug/bee**	**Oral LD_50_ ug/bee**	**Limit of detection (ppb)**
3-keto-carbofuran	Metabolite of carbofuran			2
3-OH-carbofuran	Metabolite of carbofuran			2
5-OH-Imidacloprid	Metabolite of Imidacloprid		0.159 ^a^	5
Acephate	Insecticide	1.2		5
Alachlor	Herbicide	>36.2		2
Atrazine	Herbicide	>97		0.5
Azinphos-methyl	Insecticide	0.42	0.15	2
Azoxystrobin	Fungicide	>200		1
Bentazon	Herbicide	>200 ^b^	>200 ^b^	2
Boscalid	Fungicide	>200	>166	1
Bromacil	Herbicide	>11		1
Carbaryl	Insecticide	1.1		2
Carbendazim	Fungicide and metabolite of benomyl and thiophanate-methyl	>50		1
Carbofuran	Insecticide	0.16		1
Chlorpyrifos	Insecticide	0.01	0.25	2
Clothianidin	Insecticide and Metabolite of Thiamethoxam	0.0439	0.00368	2
Coumaphos	Insecticide/Acaricide	24 ^c^		1
Coumaphos Oxon	Metabolite of Coumaphos			1
Cyproconazole	Fungicide	>100	>1000	20
Cyprodinil	Fungicide	>784		3
Diazinon	Insecticide	0.22	0.2	0.5
Dichlorvos	Insecticide	0.5		1
Difenconazole	Fungicide	>101	>177	1
Dimethoate	Insecticide	0.16	0.056	1
Dimethomorph	Fungicide	>10		1
Dinotefuran	Insecticide	0.047	0.023	2
Diphenylamine	Anti-oxidant	ND		10
Dithiopyr	Herbicide	81		1
Diuron	Herbicide	>145		3
Fenbuconazole	Fungicide	292		2
Fenhexamid	Fungicide	>215		5
Fenpropathrin	Insecticide	<0.1 lbs^d^ ai/acre ^d^		10
Fenthion	Insecticide	0.308		2
Fipronil	Insecticide	0.00593 ^b^	0.00417 ^b^	1
Fluvalinate	Insecticide	0.2		5
Imazalil	Fungicide	39 ^b^	35.1 ^b^	1
Imidacloprid olefin	Metabolite of Imidacloprid		0.036 ^a^	10
Imidacloprid urea	Metabolite of Imidacloprid		99.5 ^a^	3
Imidacloprid	Insecticide	0.0439	0.0039	1
Indoxacarb	Insecticide	0.118	18.52	10
Indoxacarb	Insecticide	0.07 ^b^	0.194 ^b^	10
Malathion	Insecticide	0.2	0.38	2
Metalaxyl	Fungicide	>100		1
Methamidophos	Insecticide	1.37		10
Methiocarb	Insecticide	0.375		1
Methomyl	Insecticide	0.16	0.29	2
Metolachlor	Herbicide	>110	>110	0.5
Myclobutanil	Fungicide	362^b^		2
Napropamide	Herbicide		>113.5	1
Oxadiazon	Herbicide	>25		3
Oxyflourfen	Herbicide	>100		2
Pendimethalin	Herbicide	49.8		5
Phosmet	Insecticide	1.06	0.37 ^b^	1
Pinoxaden	Herbicide	>200	>100	1
Pirimicarb	Insecticide	12.56	3.01	0.5
Procymidone	Fungicide	ND		30
Prodiamine	Herbicide	>100		5
Propiconazole	Fungicide	>25		1
Propoxur	Insecticide	1.35		1
Propyzamide	Herbicide	>181		5
Pyraclostrobin	Fungicide	>100		1
Pyrimethanil	Fungicide	100	100	10
Simazine	Herbicide	96.7		1
Sulfometuron- methyl	Herbicide	100		10
Thiabendazole	Fungicide	4 ^b^	>34 ^b^	1
Thiacloprid	Insecticide	37.83	17.32	1
Thiamethoxam	Insecticide	0.024	0.005	1
Thiophanate-methyl	Fungicide	100		2
Trichlorfon	Insecticide	59.8		2
Trifloxystrobin	Fungicide	200		1

^a^ Oral LD_50_ for metabolites of imidacloprid from Nauen et al. [8].

^b^ LD_50_ for bentazon, fipronil, imazalil, mycobutanil, and thiabendazole were not in the US EPA database and were obtained from the Agritox database [6]. For indoxacarb, contact and oral LD_50_ information from both the US EPA and Agritox are presented because they were substantially different. For phosmet, contact LD_50_ information is presented from both sources, and oral LD_50_ was found in the Agritox database only.

^c^ Dahlgren et al. [7], assuming a weight of 100 mg. per worker bee

^d^ Field study was the only data available in US EPA Ecotox database.

LD_50_ information from the Pesticide Ecotoxicity Database of the Office of Pesticide Programs, Ecological Fate and Effects Division, of the U.S. Environmental Protection Agency [5], unless otherwise noted.

A list of pesticides found, their uses, available information on contact and oral LD_50_ for honey bees, and analytical limits of detection are presented in Table 1. The LD_50_ values in the Ecotox and Agritox databases were typically equivalent, but we found some differences, notably for indoxacarb and phosmet, so both sets of values are presented here.

Note that the LD_50_ values range widely. As would be expected, insecticides are generally more toxic to honey bees than fungicides or herbicides, but even among the insecticides, the contact LD_50_ values range from 0.0059 μg/bee for fipronil to 59.8 μg/bee for trichlorfon. Oral LD_50_ values have been determined for fewer insecticides, but they range from 0.00368 μg/bee for clothianidin to 17.32 μg/bee for thiacloprid. For fungicides, the lowest contact LD_50_ was 4 μg/bee for thiabendazole, with values for many of the less toxic fungicides and herbicides reported in these databases only as greater than some threshold value.

The maximum residue concentration we found for each pesticide in any single sample is given in Table 2, along with summary statistics over all sites and samples. Surprisingly, given our sampling in representative sites rather than sites where pesticide overuse was suspected, we found maximum residue concentrations of some pesticides higher than the maximum levels reported in Johnson et al. [4], a review paper compiling maximum concentrations over 11 studies of pesticide residues in pollen, including some following colonies with suspected pesticide problems. Specifically, the maximum residue of phosmet reported here is 39X the maximum residue in Johnson et al. [4], carbendazim is 12X higher, myclobutanil is 4.2X higher, and indoxacarb is 1.2X higher. For other pesticides, such as carbaryl, fluvalinate, and coumaphos, the studies reviewed in Johnson et al. [4] have found residue concentrations hundreds to thousands of times higher than we report here. 

**Table 2:** Maximum pesticide residues found in trapped pollen presented as Pollen Hazard Quotients based on ratio of maximum residue (in ppb) ÷ LD_50_ (in ug/bee), and statistics on number of detections and range and variability of residue concentrations in ppb.

**Pesticide**	**Maximum PHQ contact**	**Maximum PHQ oral**	**No. detections (of 313 samples)**	**% of samples with detections**	**Maximum (ppb)**	**Minimum (ppb)**	**Median (ppb)**	**90th %tile (ppb**)	**Mean (ppb)**	**Standard deviation (ppb)**
Phosmet	75,255	44,746^a^	103	32.90	16556	1	3.7	63.8	226.5	1672.8
Imidacloprid	1,595	17,949	38	12.10	70	1	2.8	7.3	5.2	11.3
Indoxacarb	5,957^a^	2,149^a^	4	1.30	417	39	198	396	213	197.3
Chlorpyrifos	2,520	101	14	4.50	25.2	2	4.4	11.6	6.8	6.2
Fipronil	590	839	2	0.60	3.5	2	2.8	3.4	2.8	1.1
Thiamethoxam	171	820	3	1.00	4.1	1.5	2.9	3.9	2.8	1.3
Azinphos-methyl	290	813	5	1.60	122	5	7.8	79.6	31.2	51
Fenthion	640		16	5.10	197	2.6	20	103.5	41.1	53.9
Dinotefuran	162	330	3	1.00	7.6	2.1	2.3	6.5	4	3.1
Carbaryl	206		127	40.60	227	2	13	58.2	27.7	39
Fluvalinate	200		1	0.30	40	40	40	40	40	
Methomyl	150	83	12	3.80	24	2.2	8	19.6	10.3	7.3
Diazinon	82	90	3	1.00	18	1.4	1.5	14.7	7	9.6
Malathion	67	35	2	0.60	13.4	8.9	11.2	13	11.2	3.2
Carbendazim	36		92	29.40	1800	1	5	106.6	49.8	193.8
5-OH-Imidacloprid		35	1	0.30	5.6	5.6	5.6	5.6	5.6	
Acephate	33		6	1.90	40	6	10.1	39	18.9	15.6
Dimethoate	26	75	4	1.30	4.2	1.1	1.9	3.6	2.3	1.4
Dichlorvos	19		2	0.60	9.4	4.2	6.8	8.9	6.8	3.7
Carbofuran	18		2	0.60	2.8	2.3	2.6	2.8	2.6	0.4
Methamidophos	16		1	0.30	22	22	22	22	22	
Thiophanate-methyl	14		28	8.90	1413	3.1	13	279.3	110.9	276.5
Myclobutanil	12		10	3.20	4190	2.2	50	1733	611.3	1334.7
Dimethomorph	6.9		13	4.20	69	1.2	4.9	54	19.8	24
Coumaphos	6.79		146	46.60	163	1	3.5	10.6	5.8	13.7
Propoxur	5.56		1	0.30	7.5	7.5	7.5	7.5	7.5	
Boscalid	4.24	5.1	24	7.70	848	1	3.4	21.9	42.1	171.8
Pendimethalin	3.96		26	8.30	197	5.5	17	74.5	32.8	42
Methiocarb	3.73		1	0.30	1.4	1.4	1.4	1.4	1.4	
Alachlor	3.43		3	1.00	124	5.2	15	102.2	48.1	65.9
Dithiopyr	2.46		58	18.50	199	1	3.6	8.8	8.9	27.2
Thiacloprid	1.8	3.9	4	1.30	68	1	10.1	51.2	22.3	30.8
Fenbuconazole	1.36		7	2.20	396	6.1	21	232.2	91.7	140.4
Thiabendazole	1.03	0.12	3	1.00	4.1	1.1	1.3	3.5	2.2	1.7
Atrazine	0.91		84	26.80	88	0.5	1	3.8	2.8	9.7
Fenhexamid	0.85		3	1.00	182	17	105	166.6	101.3	82.6
Bromacil	0.85		3	1.00	9.3	3.2	4	8.2	5.5	3.3
Trifloxystrobin	0.8		9	2.90	160	1	6.3	52	25.3	51.3
Pyraclostrobin	0.67		5	1.60	67	2.1	6.8	45.3	19.1	27.1
Simazine	0.53		14	4.50	51	1.1	4.9	29.8	11.1	14.7
Pyrimethanil	0.52	0.52	5	1.60	52	10	25	41.6	27.4	15.2
Propyzamide	0.52		2	0.60	94	72	83	91.8	83	15.6
Sulfometuron- methyl	0.37		1	0.30	37	37	37	37	37	
Propiconazole	0.29		3	1.00	7.3	1.8	2.4	6.3	3.8	3
Azoxystrobin	0.28		17	5.40	55	1	1.8	16.8	7.5	13.5
Napropamide		0.26	10	3.20	29.7	1	2.5	14.4	6.3	8.9
Oxadiazon	0.25		1	0.30	6.2	6.2	6.2	6.2	6.2	
Trichlorfon	0.23		1	0.30	14	14	14	14	14	
Oxyflourfen	0.18		2	0.60	18	3.7	10.9	16.6	10.9	10.1
Difenconazole	0.18	0.1	6	1.90	18	3.9	11	17	10.9	6.1
Prodiamine	0.1		1	0.30	9.5	9.5	9.5	9.5	9.5	
Metalaxyl	0.09		8	2.60	8.8	1.9	3.6	6.7	4.2	2.4
Metolachlor	0.06	0.06	6	1.90	6.8	0.5	1	4.6	2.1	2.4
Cyprodinil	0.05		6	1.90	37	4.2	10.7	34	16.6	14
Bentazon	0.04	0.04	2	0.60	7.2	2.5	4.9	6.7	4.9	3.3
Imazalil	0.03	0.03	1	0.30	1	1	1	1	1	
Coumaphos Oxon			7	2.20	27	1	1.8	12.8	5.4	9.6
Fenpropathrin			3	1.00	94	33	54	86	60.3	31
3-keto-carbofuran			2	0.60	20	11	15.5	19.1	15.5	6.4
3-OH-carbofuran			2	0.60	8.4	5.2	6.8	8.1	6.8	2.3

a Based on LD_50_ from Agritox database [6].

When we use the Pollen Hazard Quotient (PHQ) to characterize these maximum residue concentrations in relation to LD_50_ values (Table 2), we find that a fairly low absolute concentration of an insecticide highly toxic to bees, such as fipronil (3.5 ppb), are more important relative to LD_50_ than a much higher absolute concentration of carbaryl (227 ppb), which has an LD_50_ >180 X higher. 

We have presented in the tables S1-S5, broken down by site and year, the number of samples in which each pesticide was detected, the maximum residue concentration in parts per billion (ppb), and the calculated maximum PHQ based on the contact LD_50_, since oral LD_50_ values are available for relatively few pesticides. 

Some pesticides were found consistently in all sites and in nearly all years (Tables S1, S2, S3, S4, S5). For example, even though neither beekeeper had used the acaricide coumaphos in several years, and even though we were measuring pesticide residues in pollen trapped as bees were entering the hive, rather than pollen stored in the hive in contact with wax left over from previous years, we still had low but detectable levels of coumaphos in the pollen in every site and in every year at every site except 2010 in Farmington. This residue is presumably due to small amounts of coumaphos volatilizing within the hive and redepositing on the pollen in the pollen trap. The insecticides carbaryl and imidacloprid, the fungicide and metabolite carbendazim, and the herbicides atrazine and pendimethalin were also found in all sites (Tables S1-S5). 

In Table 3, we present for each pesticide the relationship of the maximum residue concentration found to the percentage of the LD_50_ this would represent for a nurse bee consuming pollen at the maximum rate (9.5 mg. per day [11]). The insecticide phosmet had the highest concentration, 16556 ppb, also the highest PHQ values. The PHQ based on the oral LD_50_, 45746, would correspond to a maximum daily exposure to 42.5% of the oral LD_50_ based on these assumptions. 

**Table 3:** Relationship of the maximum residue detected in a sample of trapped pollen to the contact and oral LD_50_ for an adult worker honey bee, based on a consumption of 9.5 mg of pollen per bee per day (for nurse bees, [11]).

**Pesticide**	**Maximum residue (ppb**)	**Maximum amount ingested per nurse bee per day (ng ai)**	**Percentage of contact LD_50_**	**Percentage of oral LD_50_**
Phosmet^a^	16556	157.282	71.49	42.51
Imidacloprid	70	0.665	1.51	17.05
Indoxacarb^a^	417	3.962	5.66	2.04
Fipronil	3.5	0.033	0.56	0.80
Thiamethoxam	4.1	0.039	0.16	0.78
Dinotefuran	7.6	0.072	0.15	0.31
Chlorpyrifos	25.2	0.239	2.39	0.10
Diazinon	18	0.171	0.08	0.09
Methomyl	24	0.228	0.14	0.08
Dimethoate	4.2	0.04	0.02	0.07
Azinphos-methyl	7.8	0.074	0.02	0.05
Malathion	13.4	0.127	0.06	0.03
5-OH-Imidacloprid	5.6	0.053		0.03
Fenthion	197	1.872	0.61	
Carbaryl	227	2.157	0.20	
Fluvalinate	40	0.38	0.19	
Carbendazim	1800	17.1	0.03	
Acephate	40	0.38	0.03	
Methamidophos	22	0.209	0.02	
Dichlorvos	9.4	0.089	0.02	
Carbofuran	2.8	0.027	0.02	
Myclobutanil	4190	39.805	0.01	
Thiophanate-methyl	1413	13.424	0.01	
Coumaphos	163	1.549	0.01	
Dimethomorph	69	0.656	0.01	
Propoxur	7.5	0.071	0.01	

a Based on LD_50_ from Agritox database [6].

All pesticides with percentage of both contact and oral LD_50_ below 0.01% were omitted.

We can then use these PHQ levels to screen all of the detections of pesticides to analyze the frequency of exposure at residue concentrations corresponding to this level of hazard. These data with frequencies over all sites and years, are presented in Table 4. Many of the pesticides had one or a few spikes and many detections at lower levels. Thus, even though phosmet had by far the highest absolute concentration and the highest PHQ value in any single sample and was detected 103 times, only 24 had an PHQ over 50, 9 detections had an PHQ over 500 based on the contact LD_50_ and, based on the higher oral LD_50,_ there were 20 detections over PHQ 50 and 4 over PHQ 500. Imidacloprid also had a pattern of a few spikes and many detections at lower levels, but because the oral LD_50_ level is so low, all the detections were at an PHQ over 50, and 21 were at an PHQ level over 500. Imidacloprid residues with PHQ >50 were widespread in all 5 sites, and PHQ levels > 500 in samples in 4 sites.

**Table 4:** The total number of sites (out of 5) and detections (out of a total of 313 samples analyzed from all sites and years) with residue concentrations for which HQ >50 using LD_50_ values for either oral or contact toxicity.

**Pesticide**	**PHQ based on contact or oral LD_50_**	**Concentration in ppb forPHQ = 50**	**Sites with with PHQ > 50**	**Total detections**	**Samples with PHQ > 50**	**Samples with PHQ > 500**
Phosmet	Oral^a^	18.5	2	103	20	4
	Contact	11.0	4		24	9
Imidacloprid	Oral	0.195	5	38	38	21
	Contact	2.2	4		20	1
Indoxacarb	Oral^a^	9.7	1	4	4	2
	Contact^a^	3.5	1		4	4
Chlorpyrifos	Oral	12.5	1	14	1	0
	Contact	5.0	4		23	4
Fipronil	Oral	0.21	1	2	2	1
	Contact	0.23	1		2	1
Thiamethoxam	Oral	0.25	3	3	4	2
	Contact	1.2	2		3	0
Azinphos-methyl	Oral	7.5	1	5	3	1
	Contact	21	1		1	0
Fenthion	Contact	15.4	2	16	8	1
Dinotefuran	Oral	1.15	1	3	4	0
	Contact	2.35	1		1	0
Carbaryl	Contact	55.0	4	127	14	0
Fluvalinate	Contact	10.0	1	1	1	0
Methomyl	Oral	14.5	2	12	4	0
	Contact	8.0	2		6	0
Diazinon	Oral	10.0	1	3	1	0
	Contact	11.0	1		1	0
Malathion	Oral	19.0	0	2	0	0
	Contact	10.0	1		1	0

a Based on LD_50_ from Agritox database [6].

Chlorpyrifos, like phosmet, has a lower contact than oral LD_50_, and, based on that PHQ, residues with PHQ > 50 were widespread, in 4 sites. Although carbaryl has a high LD_50_ compared to the above insecticides, it also had residues with PHQ >50 in 4 of the 5 sites. By contrast, indoxacarb had a high maximum PHQ, particularly using the lower contact LD_50_, but was narrowly distributed with all residues detected from a single site in a single year. 

We recognize that there are a number of assumptions in using the LD_50_, a standard measured under laboratory conditions quite different from the realities of honey bee exposure, to evaluate the importance of residues found in pollen collected by honey bee colonies in the field. The contact LD_50_ is measured by applying the active ingredient of the pesticide in a solvent directly to the exoskeleton of the bee, and the oral LD_50_ is measured by feeding the active ingredient in a solution of sugar water, not pollen, and both are strictly laboratory measurements made on caged adult worker bees [16]. 

A host of other potential effects on honey bee colonies are not addressed by this method, and pesticide regulators are putting in place standardized methods to address some of these effects in a tiered protocol [16], including potential effects on survival and healthy development of larvae, the stage that consumes most of the pollen [15]. Sublethal doses of thiamethoxam, acetamiprid and fipronil can also affect behavior of adult honey bees at chronic doses from 1/5 to 1/500 of LD_50_ , depending on the mode of action of the pesticide and whether it is administered orally or by contact [18]. In addition, some combinations of pesticides, including fungicides with insecticides, have the potential to act synergistically to increase toxicity to bees [19]. 

We do not want to minimize the importance of research into other possible effects of pesticides that are not captured in acute oral or contact LD_50_ values as measured on adult worker bees. Instead, we want to make sure that scientists utilize the available information to communicate to beekeepers and farmers at least one aspect of pesticide exposure of bees – the relationship of the residues we find to the values that have been measured to kill 50% of the adult workers under laboratory conditions. The concept of Hazard Quotients can be expanded to other matrices – nectar, honey, and wax for example. With additional research on the toxicology of pesticides to different aspects of honey bee biology, this concept could also be expanded using additional measurements of LD_50_ – for pollen, an LD_50_ for larvae would be particularly valuable, since this stage is likely to be most directly affected by pesticide residues in pollen [15].

Relating the Hazard Quotient values for different matrices directly to percentages of LD_50_ values provides an additional step toward making both pesticide residue concentrations and Hazard Quotient values more meaningful. As in the examples here, information on maximum consumption of pollen at a particular honey bee life stage can be used to calculate a percentage of the LD_50_ represented by a Hazard Quotient, and then screening the residue concentration for that Hazard Quotient level allows us to describe our findings in terms that are simple to grasp: the number of sites with concentrations above a certain hazard level, and the frequency of samples above that level, both by year and by number of samples within a year. 

This concept, too, could be extended to other matrices, for example nectar. According to Rortais et al. [15], nectar foraging bees have the greatest daily consumption of sugar (in the form of nectar), consuming 32 - 128 mg of sugar/bee/day. Using the mean of this range (80 mg) and using a mean sugar content of 35% (average for squash nectar [20]), the nectar foraging bees would consume 229 mg of nectar per day. This is 24 X the maximum amount of pollen consumed per day (9.5 mg [11]), so a nectar foraging bee consuming nectar with 35% sugar content and Nectar Hazard Quotient of 50 (calculated the same way as the Pollen Hazard Quotient above, pesticide concentration as ppb ÷ LD_50_ as ug/bee), would consume 1.1% of the LD_50_ per day.

Applying this concept of Nectar Hazard Quotient to the example of the mean level of 10 ppb imidacloprid in squash nectar after soil treatment in a previous study [21], the Nectar Hazard Quotient would be 2564, and a nectar foraging bee consuming 229 mg of nectar would consume 59% of the oral LD_50_ for imidacloprid per day.

Presenting pesticide residue data as Hazard Quotients, choosing meaningful Hazard Quotient levels for each matrix that represent an easily understood relationship to the LD_50,_ and then evaluating the frequency with which pesticide residues in that matrix exceed those Hazard Quotient levels, will contribute to clearer communication among scientists and to beekeepers and the general public about the risks posed to honey bees by their exposure to pesticide residues.

## Conclusions

1. Presenting Pollen Hazard Quotient values for pesticide residues uses the available oral and contact LD_50_ data from regulatory agencies to screen pesticide concentrations relative to acute toxicity to honey bees. Using measurements of maximum pollen consumption per bee per day, PHQ values can be related to a percentage of the LD_50_ that would be consumed per bee per day.
2. Using this approach on pesticide residues in pollen trapped from honey bee colonies in 5 representative locations in Connecticut, and using the lower of the oral or contact LD_50_ to calculate the PHQ, we found that imidacloprid was the pesticide most frequently detected at PHQ > 50 (38 detections in all 5 sites) and at PHQ > 500 (21 detections at 4 sites). Phosmet had the highest absolute PHQ value (75255 PHQ contact), and phosmet, chlorpyrifos, and carbaryl were also frequently detected at PHQ > 50 (24 detections at 4 sites, 23 detections at 4 sites, and 14 detections at 4 sites, respectively). Indoxacarb had a high maximum PHQ value, but was found above PHQ > 50 only 4 times, all in a single site and a single year.
3. The concept of Hazard Quotients can be extended to other matrices. Because the maximum daily consumption of nectar is about 24X higher than the maximum daily consumption of pollen, a particular value of Nectar Hazard Quotient represents a 24X higher percentage of the LD_50_ than the equivalent Pollen Hazard Quotient.

## Supporting Information

Table S1 **Farmington**. Count of number of detections (of the total samples analyzed), maximum residue measured (in ppb), and the maximum Pollen Hazard Quotient = maximum residue (ppb) ÷ contact LD_50_ (ug/bee) for each year of sampling and over all years.
(DOCX)

Table S2 **New Haven.** Count of number of detections (of the total samples analyzed), maximum residue measured (in ppb), and the Maximum Pollen Hazard Quotient = maximum residue (ppb) ÷ contact LD_50_ (ug/bee) for each year of sampling and over all years.
(DOCX)

Table S3 **Hamden**. Count of number of detections (of the total samples analyzed), maximum residue measured (in ppb), and the Maximum Pollen Hazard Quotient = maximum residue (ppb) ÷ contact LD_50_ (ug/bee) for each year of sampling and over all years.
(DOCX)

Table S4 **Cheshire**. Count of number of detections (of the total samples analyzed), maximum residue measured (in ppb), and the Maximum Pollen Hazard Quotient = maximum residue (ppb) ÷ contact LD_50_ (ug/bee) for each year of sampling and over all years.
(DOCX)

Table S5 **Ellington**. Count of number of detections (of the total samples analyzed), maximum residue measured (in ppb), and the Maximum Pollen Hazard Quotient = maximum residue (ppb) ÷ contact LD_50_ (ug/bee) for each year of sampling and over all years.
(DOCX)

## References

[1] vanEngelsdorp D, Caron D, Hayes J, Underwood R, Henson M et al. (2012) A national survey of managed honey bee 2010-11 winter colony losses in the USA: results from the Bee Informed Partnership. J Apic Res 51: 115-124. doi:10.3896/IBRA.1.50.1.01.

[2] vanEngelsdorp D, Meixner MD (2010) A historical review of managed honey bee populations in Europe and the United States and the factors that may affect them. J Invertebr Pathol 103: S80-S95. doi:10.1016/j.jip.2009.06.011. PubMed: 19909973. 19909973

[3] Neumann P, Carreck NL (2010) Honey bee colony losses. J Apic Res 49: 1-6. doi:10.3896/IBRA.1.49.1.01.

[4] Johnson RM, Ellis MD, Mullin CA, Frazier M (2010) Pesticides and honey bee toxicity – USA. Apidologie 41: 312-331. doi:10.1051/apido/2010018.

[5] US EPA Pesticide Ecotoxicity Database of the Office of Pesticide Programs, Ecological Fate and Effects Division. Available: http://www.ipmcenters.org/Ecotox/. Accessed 2013 March 8.

[6] Agritox Agence nationale de sécurité sanitaire de l’alimentation, de l’environnement et du travail in France. Available: http://www.dive.afssa.fr/agritox/index.php. Accessed 2013 March 8.

[7] Dahlgren L, Johnson RM, Siegfried BD, Ellis MD (2012) Comparative toxicity of acaricides to honey bee (Hymenoptera: Apidae) workers and queens. J Econ Entomol 105: 1895-1902. doi:10.1603/EC12175. PubMed: 23356051. 23356051

[8] Nauen R, Ebbinghaus-Kintscher U, Schmuck R (2001) Toxicity and nicotinic acetylcholine receptor interaction of imidacloprid and its metabolites in *Apis* *mellifera* (Hymenoptera: Apidae). Pest Manag Sci 57: 577-586. doi:10.1002/ps.331. PubMed: 11464788. 11464788

[9] Halm MP, Rortais A, Arnold G, Taséi JN, Rault S (2006) New risk assessment approach for systemic insecticides: the case for honey bees and imidacloprid (Gaucho). Environ Sci Technol 40: 2448-2454. doi:10.1021/es051392i. PubMed: 16646488. 16646488

[10] Alix A, Vergnet C (2007) Risk assessment to honey bees: a scheme developed in France for non-sprayed systemic compounds. Pest Manag Sci 63: 1069-1080. doi:10.1002/ps.1463. PubMed: 17879981. 17879981

[11] Crailsheim K, Schneider LHW, Hrassnigg N, Buhlmann G, Brosch U et al. (1992) Pollen consumption and utilization in worker honeybees (*Apis* *mellifera* *carnica*): Dependence on individual age and function. J Insect Physiol 38: 409-419. doi:10.1016/0022-1910(92)90117-V.

[12] Detroy BF, Harp ER (1976). Trapping pollen from honey bee colonies. Production Research Report No. 163 Madison, WI: Agricultural Research Service Available: http://www.beesource.com/plans/pollentrap.htm. Accessed 2013 March 22.

[13] Lehotay SJ (2005) Quick, easy, cheap, effective, rugged and safe (QuEChERS) approach for determining pesticide residues. In: Martinez Vidal JL Garrido Frenich A Methods in Biotechnology, Vol. 19, Pesticide Protocols. Totowa,NJ: Humana Press pp.239-261.

[14] Villa S, Vighi M, Finizio A, Serini GB (2000) Risk assessment for honeybees from pesticide-exposed pollen. Ecotoxicology 9: 287-297. doi:10.1023/A:1026522112328.

[15] Rortais A, Arnold G, Halm MP, Touffet-Briens F (2005) Modes of honeybees exposure to systemic insecticides: Estimated amounts of contaminated pollen and nectar consumed by different categories of bees. Apidologie 36: 71-83. doi:10.1051/apido:2004071.

[16] Fischer D, Moriarty T (2011) Pesticide risk assessment for pollinators: Summary of a SETAC Pellston workshop. Available: hhttp://c.ymcdn.com/sites/www.setac.org/resource/resmgr/publications_and_resources/executivesummarypollinators_.pdf?hhsearchterms=setac+and+pellston+and+workshop. Accessed 2013 September 13.

[17] Mullin CA, Frazier M, Frazier JL, Ashcraft S, Simonds R et al. (2010) High levels of miticides and agrochemicals in North American apiaries: implications for honey bee health. PLOS ONE 5(3): e9754. doi:10.1371/journal.pone.0009754. PubMed: 20333298. 20333298 PMC2841636

[18] Aliouane Y, el Hassani AK, Gary V, Armengaud C, Lambin M et al. (2009) Subchronic exposure of honeybees to sublethal doses of pesticides: effects on behavior. Environ Toxicol Chem 28: 113–122. doi:10.1897/08-110.1. PubMed: 18700810. 18700810

[19] Iwasa T, Motoyama N, Ambrose JT, Roe RM (2004) Mechanism for the differential toxicity of neonicotinoid insecticides in the honey bee, *Apis* *mellifera* . Crop Protect 23: 371-378. doi:10.1016/j.cropro.2003.08.018.

[20] Nepi M, Guarnieri M, Pacini E (2001) Nectar secretion, reabsorption, and sugar composition in male and female flowers of *Cucurbita* *pepo* . Int J Plant Sci 162: 353-358. doi:10.1086/319581.

[21] Stoner KA, Eitzer BD (2012) Movement of soil-applied imidacloprid and thiamethoxam into nectar and pollen of squash (*Cucurbita* *pepo*). PLOS ONE 7(6): e39114. doi:10.1371/journal.pone.0039114. PubMed: 22761727. 22761727 PMC3384620

