# Correction: Using a Hazard Quotient to Evaluate Pesticide Residues Detected in Pollen Trapped from Honey Bees (*Apis mellifera*) in Connecticut

**DOI:** 10.1371/journal.pone.0159696

**Published:** 2016-07-14

**Authors:** Kimberly A. Stoner, Brian D. Eitzer

In Table 1, there should be two lines of LD_50_ values for phosmet: one from Ecotox and one from Agritox. The contact LD_50_ for phosmet of 0.22 ug/bee and oral LD_50_ for phosmet of 0.37 ug/bee from Agritox were used for calculations in all succeeding tables. Please see the correct Table 1 and its caption here.

**Table 1:** Pesticides detected in pollen trapped by honey bee hives, with pesticide use and information on acute toxicity to adult worker honey bees.

Pesticide	Use	Contact LD_50_ ug/bee	Oral LD_50_ ug/bee	Limit of detection (ppb)
3-keto-carbofuran	Metabolite of carbofuran			2
3-OH-carbofuran	Metabolite of carbofuran			2
5-OH-Imidacloprid	Metabolite of Imidacloprid		0.159 ^a^	5
Acephate	Insecticide	1.2		5
Alachlor	Herbicide	>36.2		2
Atrazine	Herbicide	>97		0.5
Azinphos-methyl	Insecticide	0.42	0.15	2
Azoxystrobin	Fungicide	>200		1
Bentazon	Herbicide	>200^b^	>200 ^b^	2
Boscalid	Fungicide	>200	>166	1
Bromacil	Herbicide	>11		1
Carbaryl	Insecticide	1.1		2
Carbendazim	Fungicide and metabolite of benomyl and thiophanate-methyl	>50		1
Carbofuran	Insecticide	0.16		1
Chlorpyrifos	Insecticide	0.01	0.25	2
Clothianidin	Insecticide and Metabolite of Thiamethoxam	0.0439	0.00368	2
Coumaphos	Insecticide/Acaricide	24 ^c^		1
Coumaphos Oxon	Metabolite of Coumaphos			1
Cyproconazole	Fungicide	>100	>1000	20
Cyprodinil	Fungicide	>784		3
Diazinon	Insecticide	0.22	0.2	0.5
Dichlorvos	Insecticide	0.5		1
Difenconazole	Fungicide	>101	>177	1
Dimethoate	Insecticide	0.16	0.056	1
Dimethomorph	Fungicide	>10		1
Dinotefuran	Insecticide	0.047	0.023	2
Diphenylamine	Anti-oxidant	ND		10
Dithiopyr	Herbicide	81		1
Diuron	Herbicide	>145		3
Fenbuconazole	Fungicide	292		2
Fenhexamid	Fungicide	>215		5
Fenpropathrin	Insecticide	<0.1 lbs^d^ ai/acre ^d^		10
Fenthion	Insecticide	0.308		2
Fipronil	Insecticide	0.00593 ^b^	0.00417 ^b^	1
Fluvalinate	Insecticide	0.2		5
Imazalil	Fungicide	39 ^b^	35.1 ^b^	1
Imidacloprid olefin	Metabolite of Imidacloprid		0.036 ^a^	10
Imidacloprid urea	Metabolite of Imidacloprid		99.5 ^a^	3
Imidacloprid	Insecticide	0.0439	0.0039	1
Indoxacarb	Insecticide	0.1180.07 ^b^	18.520.194 ^b^	10
Indoxacarb	Insecticide	0.07 ^b^	0.194 ^b^	10
Malathion	Insecticide	0.2	0.38	2
Metalaxyl	Fungicide	>100		1
Methamidophos	Insecticide	1.37		10
Methiocarb	Insecticide	0.375		1
Methomyl	Insecticide	0.16	0.29	2
Metolachlor	Herbicide	>110	>110	0.5
Myclobutanil	Fungicide	362^b^		2
Napropamide	Herbicide		>113.5	1
Oxadiazon	Herbicide	>25		3
Oxyflourfen	Herbicide	>100		2
Pendimethalin	Herbicide	49.8		5
Phosmet	Insecticide	1.060.22 ^b^		1
Phosmet	Insecticide	0.22^b^	0.37 ^b^	1
Pinoxaden	Herbicide	>200	>100	1
Pirimicarb	Insecticide	12.56	3.01	0.5
Procymidone	Fungicide	ND		30
Prodiamine	Herbicide	>100		5
Propiconazole	Fungicide	>25		1
Propoxur	Insecticide	1.35		1
Propyzamide	Herbicide	>181		5
Pyraclostrobin	Fungicide	>100		1
Pyrimethanil	Fungicide	100	100	10
Simazine	Herbicide	96.7		1
Sulfometuron- methyl	Herbicide	100		10
Thiabendazole	Fungicide	4 ^b^	>34 ^b^	1
Thiacloprid	Insecticide	37.83	17.32	1
Thiamethoxam	Insecticide	0.024	0.005	1
Thiophanate-methyl	Fungicide	100		2
Trichlorfon	Insecticide	59.8		2
Trifloxystrobin	Fungicide	200		1

^a^ Oral LD_50_ for metabolites of imidacloprid from Nauen et al. [8].

^b^ LD_50_ for bentazon, fipronil, imazalil, mycobutanil, and thiabendazole were not in the US EPA database and were obtained from the Agritox database [6]. For indoxacarb, contact and oral LD_50_ information from both the US EPA and Agritox are presented because they were substantially different. For phosmet, contact LD_50_ information is presented from both sources, and oral LD_50_ was found in the Agritox database only.

^c^ Dahlgren et al. [7], assuming a weight of 100 mg. per worker bee

^d^ Field study was the only data available in US EPA Ecotox database.

LD_50_ information from the Pesticide Ecotoxicity Database of the Office of Pesticide Programs, Ecological Fate and Effects Division, of the U.S. Environmental Protection Agency [5], unless otherwise noted.
