# Supplementary material for: Using a Hazard Quotient to Evaluate Pesticide Residues Detected in Pollen Trapped from Honey Bees (Apis mellifera) in Connecticut
Source: PLoS One. 2013 Oct 15;8(10):e77550. doi: 10.1371/journal.pone.0077550 (PMC3797043; doi:10.1371/journal.pone.0077550)
Supplement: Table S1 — Farmington. Count of number of detections (of the total samples analyzed), maximum residue measured (in ppb), and the maximum Pollen Hazard Quotient = maximum residue (ppb) ÷ contact LD50 (ug/bee) for each year of sampling and over all years. (DOCX) [file pone.0077550.s001.docx]

Table S1. Farmington. Count of number of detections (of the total samples analyzed), maximum residue measured (in ppb), and the maximum Pollen Hazard Quotient = maximum residue (ppb) ÷ contact LD_50_ (ug/bee) for each year of sampling and over all years. When no contact LD_50_ for the compound was available, the cell for Max PHQ contact was left blank. Contact LD_50_ values are from the sources cited in Table 1.

|  | **Over all years** | | | **2007** | | | **2008** | | | **2009** | | | **2010** | | |
| --- | --- | --- | --- | --- | --- | --- | --- | --- | --- | --- | --- | --- | --- | --- | --- |
| **Pesticide** | **Count (of 69)** | **Max. (ppb)** | **Max PHQ contact** | **Count (of 32)** | **Max. (ppb)** | **Max PHQ contact** | **Count (of 9)** | **Max. (ppb)** | **Max PHQ contact** | **Count (of 15)** | **Max. (ppb)** | **Max PHQ contact** | **Count (of 13)** | **Max (ppb)** | **Max PHQ contact** |
| 3-Keto-carbofuran | 2 | 20 | - | 0 | 0 | - | 0 | 0 | 0.00 | 0 | 0 | - | 2 | 20 | - |
| Acephate | 1 | 6 | 5.00 | 0 | 0 | 0.00 | 0 | 0 | 0.00 | 0 | 0 | 0.00 | 1 | 6 | 5.00 |
| Atrazine | 27 | 15 | 0.15 | 10 | 8.4 | 0.09 | 3 | 4 | 0.00 | 10 | 15 | 0.15 | 4 | 2 | 0.02 |
| Azoxystrobin | 3 | 55 | 0.28 | 0 | 0 | 0.00 | 1 | 1.8 | 0.00 | 2 | 55 | 0.28 | 0 | 0 | 0.00 |
| Boscalid | 2 | 3.2 | 0.02 | 2 | 3.2 | 0.02 | 0 | 0 | 0.00 | 0 | 0 | 0.00 | 0 | 0 | 0.00 |
| Bromacil | 1 | 3.2 | 0.29 | 0 | 0 | 0.00 | 0 | 0 | 0.00 | 0 | 0 | 0.00 | 1 | 3.2 | 0.29 |
| Carbaryl | 36 | 227 | 206 | 24 | 227 | 206 | 3 | 49 | 44.55 | 1 | 5 | 4.18 | 8 | 36 | 32.73 |
| Carbendazim | 5 | 8.9 | 0.18 | 3 | 2 | 0.04 | 0 | 0 | 0.00 | 0 | 0 | 0.00 | 2 | 8.9 | 0.18 |
| Carbofuran | 1 | 2.3 | 14.38 | 1 | 2.3 | 14.38 | 0 | 0 | 0.00 | 0 | 0 | 0.00 | 0 | 0 | 0.00 |
| Chlorpyrifos | 1 | 2.5 | 250 | 0 | 0 | 0.00 | 0 | 0 | 0.00 | 1 | 2.5 | 250 | 0 | 0 | 0.00 |
| Coumaphos | 9 | 5.1 | 0.21 | 2 | 3.4 | 0.14 | 2 | 1.1 | 0.05 | 5 | 5.1 | 0.21 | 0 | 0 | 0.00 |
| Diazinon | 3 | 18 | 81.82 | 2 | 18 | 81.82 | 0 | 0 | 0.00 | 0 | 0 | 0.00 | 1 | 1.5 | 6.82 |
| Dimethoate | 3 | 4.2 | 26.25 | 0 | 0 | 0.00 | 0 | 0 | 0.00 | 3 | 4.2 | 26.25 | 0 | 0 | 0.00 |
| Dithiopyr | 5 | 4.8 | 0.06 | 0 | 0 | 0.00 | 0 | 0 | 0.00 | 5 | 4.8 | 0.06 | 0 | 0 | 0.00 |
| Fenthion | 11 | 197 | 640 | 11 | 197 | 640 | 0 | 0 | 0.00 | 0 | 0 | 0.00 | 0 | 0 | 0.00 |
| Imidacloprid | 10 | 70 | 1595 | 7 | 70 | 1595 | 1 | 6.2 | 141 | 1 | 5 | 114 | 1 | 3.2 | 72.89 |
| Metalaxyl | 5 | 8.8 | 0.09 | 1 | 2.1 | 0.02 | 1 | 4 | 0.04 | 3 | 8.8 | 0.09 | 0 | 0 | 0.00 |
| Methomyl | 11 | 20 | 125 | 4 | 20 | 125 | 1 | 14 | 87.50 | 4 | 8 | 51.88 | 2 | 2.5 | 15.63 |
| Metolachlor | 6 | 6.8 | 0.06 | 0 | 0 | 0.00 | 0 | 0 | 0.00 | 3 | 1 | 0.01 | 3 | 6.8 | 0.06 |
| Oxyflourfen | 1 | 3.7 | 0.04 | 1 | 3.7 | 0.04 | 0 | 0 | 0.00 | 0 | 0 | 0.00 | 0 | 0 | 0.00 |
| Pendimethalin | 2 | 8.2 | 0.16 | 1 | 6 | 0.12 | 0 | 0 | 0.00 | 1 | 8.2 | 0.16 | 0 | 0 | 0.00 |
| Phosmet^a^ | 13 | 7 | 31.82 | 8 | 7 | 31.82 | 0 | 0 | 0.00 | 4 | 4 | 17.73 | 1 | 2.4 | 10.91 |
| Pyrimethanil | 2 | 52 | 0.52 | 2 | 52 | 0.52 | 0 | 0 | 0.00 | 0 | 0 | 0.00 | 0 | 0 | 0.00 |
| Simazine | 1 | 4.9 | 0.05 | 0 | 0 | 0.00 | 0 | 0 | 0.00 | 1 | 4.9 | 0.05 | 0 | 0 | 0.00 |
| Trichlorfon | 1 | 14 | 0.23 | 1 | 14 | 0.23 | 0 | 0 | 0.00 | 0 | 0 | 0.00 | 0 | 0 | 0.00 |

^a^ Maximum Pollen Hazard Quotient based on the contact LD_50_ from Agritox database [6].
