# Supplementary material for: Using a Hazard Quotient to Evaluate Pesticide Residues Detected in Pollen Trapped from Honey Bees (Apis mellifera) in Connecticut
Source: PLoS One. 2013 Oct 15;8(10):e77550. doi: 10.1371/journal.pone.0077550 (PMC3797043; doi:10.1371/journal.pone.0077550)
Supplement: Table S2 — New Haven. Count of number of detections (of the total samples analyzed), maximum residue measured (in ppb), and the Maximum Pollen Hazard Quotient = maximum residue (ppb) ÷ contact LD50 (ug/bee) for each year of sampling and over all years. (DOCX) [file pone.0077550.s002.docx]

Table S2. New Haven. Count of number of detections (of the total samples analyzed), maximum residue measured (in ppb), and the Maximum Pollen Hazard Quotient = maximum residue (ppb) ÷ contact LD_50_ (ug/bee) for each year of sampling and over all years. When no contact LD_50_ for the compound was available, the cell for Max PHQ contact was left blank. Contact LD_50_ values are from the sources cited in Table 1.

|  | **Over all years** | | | **2007** | | | **2008** | | | **2009** | | | **2010** | | | **2011** | | |
| --- | --- | --- | --- | --- | --- | --- | --- | --- | --- | --- | --- | --- | --- | --- | --- | --- | --- | --- |
| **Pesticide** | **Count (out of 101)** | **Max. (ppb)** | **Max PHQ contact** | **Count (out of 26)** | **Max. (ppb)** | **Max PHQ contact** | **Count (out of 17)** | **Max. (ppb)** | **Max PHQ contact** | **Count (out of 16)** | **Max. (ppb)** | **Max PHQ contact** | **Count (out of 19)** | **Max. (ppb)** | **Max PHQ contact** | **Count (out of 23)** | **Max. (ppb)** | **Max PHQ contact** |
| 3-OH-Carbofuran^b^ | 2 | 8.4 |  | 0 | 0 |  | 0 | 0 |  | 0 | 0 |  | 0 | 0 |  | 2 | 8.4 |  |
| Acephate | 2 | 40 | 33.33 | 0 | 0 | 0.00 | 0 | 0 | 0.00 | 0 | 0 | 0.00 | 1 | 38 | 31.67 | 1 | 40 | 33.33 |
| Atrazine | 20 | 3 | 0.03 | 0 | 0 | 0.00 | 4 | 1 | 0.01 | 6 | 1.4 | 0.01 | 7 | 3 | 0.03 | 3 | 0.8 | 0.01 |
| Azoxystrobin | 1 | 1 | 0.01 | 0 | 0 | 0.00 | 0 | 0 | 0.00 | 0 | 0 | 0.00 | 1 | 1 | 0.01 | 0 | 0 | 0.00 |
| Boscalid | 2 | 2.2 | 0.01 | 1 | 2.2 | 0.01 | 0 | 0 | 0.00 | 1 | 1.2 | 0.01 | 0 | 0 | 0.00 | 0 | 0 | 0.00 |
| Bromacil | 1 | 9.3 | 0.85 | 1 | 9.3 | 0.85 | 0 | 0 | 0.00 | 0 | 0 | 0.00 | 0 | 0 | 0.00 | 0 | 0 | 0.00 |
| Carbaryl | 44 | 193 | 175 | 16 | 55 | 50.00 | 8 | 193 | 175.45 | 1 | 3.7 | 3.36 | 9 | 46.5 | 42.27 | 10 | 21 | 19.09 |
| Carbendazim | 42 | 1800 | 36 | 3 | 4.6 | 0.09 | 11 | 1800 | 36.00 | 6 | 48 | 0.96 | 10 | 78 | 1.56 | 12 | 360 | 7.20 |
| Chlorpyrifos | 3 | 8.8 | 880 | 0 | 0 | 0.00 | 0 | 0 | 0.00 | 0 | 0 | 0.00 | 1 | 8.8 | 880 | 2 | 7.2 | 720 |
| Coumaphos | 51 | 18.3 | 0.76 | 26 | 18.3 | 0.76 | 4 | 4 | 0.17 | 14 | 15 | 0.63 | 5 | 2.2 | 0.09 | 2 | 2.6 | 0.11 |
| Coumaphos Oxon^b^ | 5 | 2.1 |  | 0 | 0 |  | 0 | 0 |  | 5 | 2.1 |  | 0 | 0 |  | 0 | 0 |  |
| Dimethoate | 1 | 1.4 | 8.75 | 0 | 0 | 0.00 | 0 | 0 | 0.00 | 1 | 1.4 | 8.75 | 0 | 0 | 0.00 | 0 | 0 | 0.00 |
| Dinotefuran | 3 | 7.6 | 162 | 0 | 0 | 0.00 | 0 | 0 | 0.00 | 0 | 0 | 0.00 | 2 | 2.3 | 48.94 | 1 | 7.6 | 162 |
| Dithiopyr | 32 | 199 | 2.46 | 0 | 0 | 0.00 | 8 | 6 | 0.07 | 7 | 20 | 0.25 | 7 | 74 | 0.91 | 10 | 199 | 2.46 |
| Fenthion | 1 | 2.6 | 8.44 | 1 | 2.6 | 8.44 | 0 | 0 | 0.00 | 0 | 0 | 0.00 | 0 | 0 | 0.00 | 0 | 0 | 0.00 |
| Fipronil | 2 | 3.5 | 590 | 1 | 2 | 337.27 | 0 | 0 | 0.00 | 0 | 0 | 0.00 | 0 | 0 | 0.00 | 1 | 3.5 | 590 |
| Imidacloprid | 18 | 18.5 | 421 | 4 | 3.4 | 77.45 | 4 | 9.5 | 216 | 6 | 18.5 | 421 | 1 | 2.7 | 61.50 | 3 | 3.6 | 82.00 |
| 5-OH-Imidacloprid^b^ | 1 | 5.6 |  | 0 | 0 |  | 0 | 0 |  | 0 | 0 |  | 0 | 0 |  | 1 | 5.6 |  |
| Metalaxyl | 1 | 2.2 | 0.02 | 0 | 0 | 0.00 | 0 | 0 | 0.00 | 0 | 0 | 0.00 | 1 | 2.2 | 0.02 | 0 | 0 | 0.00 |
| Methamidophos | 1 | 22 | 16.06 | 0 | 0 | 0.00 | 0 | 0 | 0.00 | 0 | 0 | 0.00 | 1 | 22 | 16.06 | 0 | 0 | 0.00 |
| Methiocarb | 1 | 1.4 | 3.73 | 0 | 0 | 0.00 | 0 | 0 | 0.00 | 0 | 0 | 0.00 | 1 | 1.4 | 3.73 | 0 | 0 | 0.00 |
| Myclobutanil | 2 | 4.5 | 0.01 | 0 | 0 | 0.00 | 0 | 0 | 0.00 | 0 | 0 | 0.00 | 0 | 0 | 0.00 | 2 | 4.5 | 0.01 |
| Pendimethalin | 9 | 106 | 2.13 | 0 | 0 | 0.00 | 2 | 9 | 0.18 | 2 | 27 | 0.54 | 3 | 106 | 2.13 | 2 | 11.5 | 0.23 |
| Phosmet^a^ | 23 | 13.8 | 62.73 | 7 | 4.3 | 19.55 | 1 | 1.6 | 7.27 | 4 | 13.8 | 62.73 | 3 | 5.4 | 24.55 | 8 | 3.2 | 14.55 |
| Prodiamine | 1 | 9.5 | 0.10 | 1 | 9.5 | 0.10 | 0 | 0 | 0.00 | 0 | 0 | 0.00 | 0 | 0 | 0.00 | 0 | 0 | 0.00 |
| Propiconazole | 2 | 7.3 | 0.29 | 0 | 0 | 0.00 | 0 | 0 | 0.00 | 1 | 7.3 | 0.29 | 0 | 0 | 0.00 | 1 | 1.8 | 0.07 |
| Propoxur | 1 | 7.5 | 5.56 | 0 | 0 | 0.00 | 0 | 0 | 0.00 | 0 | 0 | 0.00 | 1 | 7.5 | 5.56 | 0 | 0 | 0.00 |
| Sulfometuron-methyl | 1 | 37 | 0.37 | 0 | 0 | 0.00 | 1 | 37 | 0.37 | 0 | 0 | 0.00 | 0 | 0 | 0.00 | 0 | 0 | 0.00 |
| Thiamethoxam | 1 | 4.1 | 171 | 0 | 0 | 0.00 | 0 | 0 | 0.00 | 0 | 0 | 0.00 | 1 | 4.1 | 171 | 0 | 0 | 0.00 |
| Thiophanate-methyl | 22 | 1413 | 14.13 | 0 | 0 | 0.00 | 5 | 384 | 3.84 | 4 | 52 | 0.52 | 4 | 10 | 0.10 | 9 | 1413 | 14.13 |
|  |  |  |  |  |  |  |  |  |  |  |  |  |  |  |  |  |  |  |
|  |  |  |  |  |  |  |  |  |  |  |  |  |  |  |  |  |  |  |

^a^ Maximum Pollen Hazard Quotient based on the contact LD_50_ from Agritox database [6].

^b^ No contact LD_50_ available.
