# Supplementary material for: Using a Hazard Quotient to Evaluate Pesticide Residues Detected in Pollen Trapped from Honey Bees (Apis mellifera) in Connecticut
Source: PLoS One. 2013 Oct 15;8(10):e77550. doi: 10.1371/journal.pone.0077550 (PMC3797043; doi:10.1371/journal.pone.0077550)
Supplement: Table S3 — Hamden. Count of number of detections (of the total samples analyzed), maximum residue measured (in ppb), and the Maximum Pollen Hazard Quotient = maximum residue (ppb) ÷ contact LD50 (ug/bee) for each year of sampling and over all years. (DOCX) [file pone.0077550.s003.docx]

Table S3. Hamden. Count of number of detections (of the total samples analyzed), maximum residue measured (in ppb), and the Maximum Pollen Hazard Quotient = maximum residue (ppb) ÷ contact LD_50_ (ug/bee) for each year of sampling and over all years. When no contact LD_50_ for the compound was available, the cell for Max PHQ contact was left blank. Contact LD_50_ values are from the sources cited in Table 1.

|  | **Over all years** | | | **2007** | | | **2008** | | | **2009** | | | **2010** | | | **2011** | | |
| --- | --- | --- | --- | --- | --- | --- | --- | --- | --- | --- | --- | --- | --- | --- | --- | --- | --- | --- |
| **Pesticide** | **Count (out of 114)** | **Max. (ppb)** | **Max PHQ contact** | **Count (out of 37)** | **Max. (ppb)** | **Max PHQ contact** | **Count (out of 18)** | **Max. (ppb)** | **Max PHQ contact** | **Count (out of 16)** | **Max. (ppb)** | **Max PHQ contact** | **Count (out of 18)** | **Max. (ppb)** | **Max PHQ contact** | **Count (out of 25)** | **Max. (ppb)** | **Max PHQ contact** |
| Alachlor | 3 | 124 | 3.43 | 0 | 0 | 0.00 | 1 | 15 | 0.41 | 2 | 124 | 3.43 | 0 | 0 | 0.00 | 0 | 0 | 0.00 |
| Atrazine | 24 | 11 | 0.11 | 8 | 5.8 | 0.06 | 8 | 11 | 0.11 | 5 | 1.8 | 0.02 | 2 | 2.6 | 0.03 | 1 | 0.5 | 0.01 |
| Azinphos-methyl | 5 | 122 | 290 | 2 | 5 | 11.90 | 0 | 0 | 0.00 | 1 | 7.8 | 18.57 | 2 | 122 | 290 | 0 | 0 | 0.00 |
| Azoxystrobin | 10 | 21 | 0.11 | 1 | 1 | 0.01 | 0 | 0 | 0.00 | 2 | 21 | 0.11 | 4 | 4.1 | 0.02 | 3 | 3.3 | 0.02 |
| Bentazon | 2 | 7.2 | 0.04 | 0 | 0 | 0.00 | 0 | 0 | 0.00 | 0 | 0 | 0.00 | 2 | 7.2 | 0.04 | 0 | 0 | 0.00 |
| Boscalid | 16 | 848 | 4.24 | 1 | 848 | 4.24 | 1 | 2.2 | 0.01 | 4 | 17 | 0.09 | 5 | 24.4 | 0.12 | 5 | 16 | 0.08 |
| Bromacil | 1 | 4 | 0.36 | 0 | 0 | 0.00 | 0 | 0 | 0.00 | 0 | 0 | 0.00 | 1 | 4 | 0.36 | 0 | 0 | 0.00 |
| Carbaryl | 36 | 78 | 70.91 | 16 | 60 | 54.55 | 5 | 78 | 70.91 | 4 | 26 | 23.64 | 6 | 43 | 39.09 | 5 | 15 | 13.64 |
| Carbendazim | 35 | 200 | 4.00 | 6 | 1.8 | 0.04 | 7 | 55 | 1.10 | 9 | 74 | 1.48 | 7 | 200 | 4.00 | 6 | 108 | 2.16 |
| Carbofuran | 1 | 2.8 | 17.50 | 1 | 2.8 | 17.50 | 0 | 0 | 0.00 | 0 | 0 | 0.00 | 0 | 0 | 0.00 | 0 | 0 | 0.00 |
| Chlorpyrifos | 7 | 12.1 | 1210 | 1 | 3.9 | 390 | 2 | 4.5 | 450 | 1 | 12.1 | 1210 | 2 | 10.5 | 1050 | 1 | 4.7 | 470 |
| Coumaphos | 73 | 163 | 6.79 | 37 | 19.8 | 0.83 | 18 | 7.7 | 0.32 | 2 | 163 | 6.79 | 10 | 2.8 | 0.12 | 6 | 3.3 | 0.14 |
| Coumaphos Oxon^b^ | 1 | 27 |  | 0 | 0 |  | 0 | 0 |  | 1 | 27 |  | 0 | 0 |  | 0 | 0 |  |
| Cyprodinil | 4 | 14 | 0.02 | 0 | 0 | 0.00 | 0 | 0 | 0.00 | 0 | 0 | 0.00 | 0 | 0 | 0.00 | 4 | 14 | 0.02 |
| Difenconazole | 3 | 15 | 0.15 | 0 | 0 | 0.00 | 0 | 0 | 0.00 | 0 | 0 | 0.00 | 0 | 0 | 0.00 | 3 | 15 | 0.15 |
| Dithiopyr | 17 | 12 | 0.15 | 0 | 0 | 0.00 | 5 | 12 | 0.15 | 4 | 4.5 | 0.06 | 0 | 0 | 0.00 | 8 | 6.7 | 0.08 |
| Fenbuconazole | 5 | 396 | 1.36 | 0 | 0 | 0.00 | 0 | 0 | 0.00 | 0 | 0 | 0.00 | 1 | 12.5 | 0.04 | 4 | 396 | 1.36 |
| Fenhexamid | 1 | 17 | 0.08 | 0 | 0 | 0.00 | 1 | 17 | 0.08 | 0 | 0 | 0.00 | 0 | 0 | 0.00 | 0 | 0 | 0.00 |
| Fenthion | 4 | 26 | 84.42 | 4 | 26 | 84.42 | 0 | 0 | 0.00 | 0 | 0 | 0.00 | 0 | 0 | 0.00 | 0 | 0 | 0.00 |
| Fluvalinate | 1 | 40 | 200 | 0 | 0 | 0.00 | 0 | 0 | 0.00 | 1 | 40 | 200 | 0 | 0 | 0.00 | 0 | 0 | 0.00 |
| Imidacloprid | 9 | 7.2 | 164 | 0 | 0 | 0.00 | 4 | 7.2 | 164 | 1 | 1.9 | 43.28 | 0 | 0 | 0.00 | 4 | 4.9 | 112 |
| Indoxacarb^a^ | 4 | 417 | 5957 | 0 | 0 | 0.00 | 0 | 0 | 0.00 | 0 | 0 | 0.00 | 4 | 417 | 5957 | 0 | 0 | 0.00 |
| Malathion | 2 | 13.4 | 67.00 | 0 | 0 | 0.00 | 0 | 0 | 0.00 | 2 | 13.4 | 67.00 | 0 | 0 | 0.00 | 0 | 0 | 0.00 |
| Metalaxyl | 2 | 5.4 | 0.05 | 0 | 0 | 0.00 | 0 | 0 | 0.00 | 0 | 0 | 0.00 | 0 | 0 | 0.00 | 2 | 5.4 | 0.05 |
| Methomyl | 1 | 24 | 150 | 1 | 24 | 150 | 0 | 0 | 0.00 | 0 | 0 | 0.00 | 0 | 0 | 0.00 | 0 | 0 | 0.00 |
| Myclobutanil | 4 | 60 | 0.17 | 1 | 16 | 0.04 | 1 | 60 | 0.17 | 0 | 0 | 0.00 | 2 | 57 | 0.16 | 0 | 0 | 0.00 |
| Napropamide^b^ | 10 | 29.7 |  | 4 | 2.4 |  | 1 | 2.6 |  | 4 | 12.7 |  | 1 | 29.7 |  | 0 | 0 |  |
| Oxadiazon | 1 | 6.2 | 0.25 | 0 | 0 | 0.00 | 0 | 0 | 0.00 | 1 | 6.2 | 0.25 | 0 | 0 | 0.00 | 0 | 0 | 0.00 |
| Oxyflourfen | 1 | 18 | 0.18 | 1 | 18 | 0.18 | 0 | 0 | 0.00 | 0 | 0 | 0.00 | 0 | 0 | 0.00 | 0 | 0 | 0.00 |
| Pendimethalin | 10 | 87 | 1.75 | 0 | 0 | 0.00 | 3 | 87 | 1.75 | 0 | 0 | 0.00 | 5 | 39 | 0.78 | 2 | 25 | 0.50 |
| Phosmet^a^ | 54 | 16556 | 75255 | 8 | 7.9 | 35.91 | 8 | 750 | 3409 | 12 | 540 | 2455 | 9 | 113 | 514 | 17 | 16556 | 75255 |
| Propyzamide | 2 | 94 | 0.52 | 0 | 0 | 0.00 | 0 | 0 | 0.00 | 0 | 0 | 0.00 | 0 | 0 | 0.00 | 2 | 94 | 0.52 |
| Pyraclostrobin | 5 | 67 | 0.67 | 0 | 0 | 0.00 | 0 | 0 | 0.00 | 0 | 0 | 0.00 | 3 | 67 | 0.67 | 2 | 12.8 | 0.13 |
| Pyrimethanil | 3 | 25 | 0.25 | 2 | 24 | 0.24 | 1 | 25 | 0.25 | 0 | 0 | 0.00 | 0 | 0 | 0.00 | 0 | 0 | 0.00 |
| Simazine | 9 | 13 | 0.13 | 1 | 1.3 | 0.01 | 2 | 13 | 0.13 | 6 | 12 | 0.12 | 0 | 0 | 0.00 | 0 | 0 | 0.00 |
| Thiabendazole | 3 | 4.1 | 1.03 | 0 | 0 | 0.00 | 3 | 4.1 | 1.03 | 0 | 0 | 0.00 | 0 | 0 | 0.00 | 0 | 0 | 0.00 |
| Thiacloprid | 4 | 68 | 1.80 | 0 | 0 | 0.00 | 0 | 0 | 0.00 | 0 | 0 | 0.00 | 0 | 0 | 0.00 | 4 | 68 | 1.80 |
| Thiamethoxam | 2 | 2.9 | 121 | 0 | 0 | 0.00 | 0 | 0 | 0.00 | 1 | 1.5 | 62.50 | 1 | 2.9 | 121 | 0 | 0 | 0.00 |
| Thiophanate-methyl | 5 | 30 | 0.30 | 0 | 0 | 0.00 | 1 | 13 | 0.13 | 0 | 0 | 0.00 | 2 | 30 | 0.30 | 2 | 21 | 0.21 |
| Trifloxystrobin | 9 | 160 | 0.80 | 2 | 6.3 | 0.03 | 0 | 0 | 0.00 | 4 | 160 | 0.80 | 3 | 6.5 | 0.03 | 0 | 0 | 0.00 |
|  |  |  |  |  |  |  |  |  |  |  |  |  |  |  |  |  |  |  |
|  |  |  |  |  |  |  |  |  |  |  |  |  |  |  |  |  |  |  |

^a^ Maximum Pollen Hazard Quotient based on the contact LD_50_ from Agritox database [6].

^b^ No contact LD_50_ available.
