# Supplementary material for: Using a Hazard Quotient to Evaluate Pesticide Residues Detected in Pollen Trapped from Honey Bees (Apis mellifera) in Connecticut
Source: PLoS One. 2013 Oct 15;8(10):e77550. doi: 10.1371/journal.pone.0077550 (PMC3797043; doi:10.1371/journal.pone.0077550)
Supplement: Table S4 — Cheshire. Count of number of detections (of the total samples analyzed), maximum residue measured (in ppb), and the Maximum Pollen Hazard Quotient = maximum residue (ppb) ÷ contact LD50 (ug/bee) for each year of sampling and over all years. (DOCX) [file pone.0077550.s004.docx]

Table S4. Cheshire. Count of number of detections (of the total samples analyzed), maximum residue measured (in ppb), and the Maximum Pollen Hazard Quotient = maximum residue (ppb) ÷ contact LD_50_ (ug/bee) for each year of sampling and over all years. When no contact LD_50_ for the compound was available, the cell for Max PHQ contact was left blank. Contact LD_50_ values are from the sources cited in Table 1.

|  | **Over all years** | | | **2007** | | | **2009** | | |
| --- | --- | --- | --- | --- | --- | --- | --- | --- | --- |
| **Pesticide** | **Count (out of 11)** | **Max. (ppb)** | **Max PHQ contact** | **Count (out of 7)** | **Max. (ppb)** | **Max PHQ contact** | **Count (out of 4)** | **Max. (ppb)** | **Max PHQ contact** |
| Atrazine | 7 | 2.1 | 0.02 | 6 | 2.1 | 0.02 | 1 | 1.3 | 0.01 |
| Boscalid | 2 | 20 | 0.10 | 0 | 0 | 0.00 | 2 | 20 | 0.10 |
| Carbaryl | 8 | 177 | 161 | 5 | 177 | 161 | 3 | 138 | 125 |
| Carbendazim | 4 | 80 | 1.60 | 0 | 0 | 0.00 | 4 | 80 | 1.60 |
| Chlorpyrifos | 3 | 25.2 | 2520 | 2 | 25.2 | 2520 | 1 | 4.3 | 430 |
| Coumaphos | 3 | 20 | 0.83 | 1 | 2.5 | 0.10 | 2 | 20 | 0.83 |
| Coumaphos Oxon^b^ | 1 | 3.4 |  | 0 | 0 |  | 1 | 3.4 |  |
| Cyprodinil | 2 | 37 | 0.05 | 0 | 0 | 0.00 | 2 | 37 | 0.05 |
| Difenconazole | 3 | 18 | 0.18 | 0 | 0 | 0.00 | 3 | 18 | 0.18 |
| Dithiopyr | 4 | 11 | 0.14 | 0 | 0 | 0.00 | 4 | 11 | 0.14 |
| Fenbuconazole | 2 | 19 | 0.07 | 0 | 0 | 0.00 | 2 | 19 | 0.07 |
| Fenhexamid | 2 | 182 | 0.85 | 2 | 182 | 0.85 | 0 | 0 | 0.00 |
| Fenpropathrin^b^ | 3 | 94 |  | 0 | 0 |  | 3 | 94 |  |
| Myclobutanil | 4 | 4190 | 11.57 | 1 | 1460 | 4.03 | 3 | 4190 | 11.57 |
| Pendimethalin | 3 | 197 | 3.96 | 0 | 0 | 0.00 | 3 | 197 | 3.96 |
| Phosmet^a^ | 3 | 172 | 782 | 0 | 0 | 0.00 | 3 | 172 | 782 |
| Simazine | 4 | 51 | 0.53 | 0 | 0 | 0.00 | 4 | 51 | 0.53 |
| Thiophanate-methyl | 1 | 87 | 0.87 | 0 | 0 | 0.00 | 1 | 87 | 0.87 |
|  |  |  |  |  |  |  |  |  |  |
|  |  |  |  |  |  |  |  |  |  |

^a^ Maximum Pollen Hazard Quotient based on the contact LD_50_ from Agritox database [6].

^b^ No contact LD_50_ available.
