# Supplementary material for: Using a Hazard Quotient to Evaluate Pesticide Residues Detected in Pollen Trapped from Honey Bees (Apis mellifera) in Connecticut
Source: PLoS One. 2013 Oct 15;8(10):e77550. doi: 10.1371/journal.pone.0077550 (PMC3797043; doi:10.1371/journal.pone.0077550)
Supplement: Table S5 — Ellington. Count of number of detections (of the total samples analyzed), maximum residue measured (in ppb), and the Maximum Pollen Hazard Quotient = maximum residue (ppb) ÷ contact LD50 (ug/bee) for each year of sampling and over all years. (DOCX) [file pone.0077550.s005.docx]

Table S5. Ellington. Count of number of detections (of the total samples analyzed), maximum residue measured (in ppb), and the Maximum Pollen Hazard Quotient = maximum residue (ppb) ÷ contact LD_50_ (ug/bee) for each year of sampling and over all years. When no contact LD_50_ for the compound was available, the cell for Max PHQ contact was left blank. Contact LD_50_ values are from the sources cited in Table 1.

|  | **Over all years** | | | **2009** | | | **2010** | | |
| --- | --- | --- | --- | --- | --- | --- | --- | --- | --- |
| **Pesticide** | **Count**  **(out of 18)** | **Max. (ppb)** | **Max PHQ contact** | **Count**  **(out of 6)** | **Max. (ppb)** | **Max PHQ contact** | **Count**  **(out of 12)** | **Max. (ppb)** | **Max PHQ contact** |
| Acephate | 3 | 10.2 | 8.50 | 2 | 10 | 8.33 | 1 | 10.2 | 8.50 |
| Atrazine | 6 | 88 | 0.91 | 2 | 1.4 | 0.01 | 4 | 88 | 0.91 |
| Azoxystrobin | 3 | 11.6 | 0.06 | 0 | 0 | 0.00 | 3 | 11.6 | 0.06 |
| Boscalid | 2 | 3.2 | 0.02 | 2 | 3.2 | 0.02 | 0 | 0 | 0.00 |
| Carbaryl | 3 | 5.4 | 4.91 | 0 | 0 | 0.00 | 3 | 5.4 | 4.91 |
| Carbendazim | 6 | 7.5 | 0.15 | 3 | 7.5 | 0.15 | 3 | 3.8 | 0.08 |
| Coumaphos | 10 | 4.9 | 0.20 | 6 | 4.9 | 0.20 | 4 | 2.4 | 0.10 |
| Dichlorvos | 2 | 9.4 | 18.80 | 2 | 9.4 | 18.80 | 0 | 0 | 0.00 |
| Dimethomorph | 13 | 69 | 6.90 | 6 | 55 | 5.50 | 7 | 69 | 6.90 |
| Imazalil | 1 | 1 | 0.03 | 0 | 0 | 0.00 | 1 | 1 | 0.03 |
| Imidacloprid | 1 | 5.2 | 118 | 0 | 0 | 0.00 | 1 | 5.2 | 118 |
| Pendimethalin | 2 | 8.8 | 0.18 | 2 | 8.8 | 0.18 | 0 | 0 | 0.00 |
| Phosmet^a^ | 10 | 16.5 | 75.00 | 5 | 4.8 | 21.82 | 5 | 16.5 | 75.00 |
| Propiconazole | 1 | 2.4 | 0.10 | 0 | 0 | 0.00 | 1 | 2.4 | 0.10 |
|  |  |  |  |  |  |  |  |  |  |
|  |  |  |  |  |  |  |  |  |  |

^a^ Maximum Pollen Hazard Quotient based on the contact LD_50_ from Agritox database [6].
